# Adaptation of the Autism Spectrum Screening Questionnaire (ASSQ) to preschool children

**DOI:** 10.1371/journal.pone.0199590

**Published:** 2018-07-10

**Authors:** Masaki Adachi, Michio Takahashi, Nobuya Takayanagi, Satomi Yoshida, Sayura Yasuda, Masanori Tanaka, Ayako Osato-Kaneda, Manabu Saito, Michito Kuribayashi, Sumi Kato, Kazuhiko Nakamura

**Affiliations:** 1 Research Center for Child Mental Development, Graduate School of Medicine, Hirosaki University, Hirosaki, Aomori, Japan; 2 Faculty of Human Studies, Aichi Toho University, Nagoya, Aichi, Japan; 3 Department of Neuropsychiatry, Graduate School of Medicine, Hirosaki University, Hirosaki, Aomori, Japan; 4 Department of Management, Faculty of Business Administration, Hokkai-Gakuen University, Sapporo, Hokkaido, Japan; 5 Department of Management and Low, Aomori Chuo Gakuin University, Aomori, Japan; Harvard Medical School, UNITED STATES

## Abstract

The Autism Spectrum Screening Questionnaire (ASSQ) is equipped with good properties for screening the broader phenotype of autistic traits, but it is standardized for a limited age range—from 7 to 16 years. To contribute to the early detection of Autism Spectrum Disorder (ASD), particularly in high functioning children with ASD, likely to cause maladjustments during school age, the present study examined psychometric properties to apply the ASSQ to a younger age. We tested parents’ ASSQ ratings for preschool children in clinical (N = 154, average age 60.77 months, range 55–72 months) and community settings (N = 1390, average age 60.53 months, range 57–68 months) in Japan. The results showed, just as in school-aged children, the ASSQ had reliability and validity as a screening instrument for preschool children in community settings. A cut-off of 7 with sensitivity of 0.93 and specificity of 0.84 is recommended for community screening. Still, based on the current study with a clinical group, an optimal cut-off score with high sensitivity and high specificity for parents’ ASSQ ratings could not be established. The clinicians should be reminded that the ASSQ is a screening instrument, not a diagnosing instrument. Also, this result suggest multi-faceted evaluation is necessary in clinical settings, for example, the addition of teachers’ ratings.

## Introduction

Autism spectrum disorder (ASD) is characterized by severe social communication deficits and stereotyped, repetitive behaviors [1]. Recognition that social difficulties characteristic of ASD can appear in many different variants, depending on language skills, general ability level, severity of symptoms, context, and coexisting disorders, has led to a dramatic increase in the number of children diagnosed with ASD. The median prevalence of autism worldwide is 0.62–0.70% [2, 3], although the latest large-scale surveys have estimated 1–2% [4–8].

Autistic traits are recognized to negatively affect poor social and functioning outcomes and reduced coping strategies at sub-clinical and clinical levels [9]. For example, difficulties in social and communication skills could increase risk of social isolation [10], which could lead to secondary depression [11]. Left untreated, depressive symptoms are likely to extend into adulthood and to reduce future prospects, such as engaging in and completing tertiary education, finding jobs, and remaining employed [12]. On the other hand, social skills of high functioning children with ASD (HF-ASD) show improvement with early intervention [13]; therefore, a screening instrument that can be applied at an earlier age is important.

Although early markers of autism can be identified in the first 2 years of life [14], the average month age at which children are diagnosed with ASD is around 53–55 months, and it can be delayed until 74–116 months in mild to moderate cases [15–18]. According to Howlin and Asgharian [19], parents of children with autism were generally aware of developmental problems by 18 months (*SD* = 12 months), while parents of children with Asperger’s syndrome noticed such problems around 30 months (*SD* = 30 months). The average age of confirmed diagnoses was 3–5 years in the autism group and 5–9 years in the Asperger’s syndrome group. Consequently, diagnoses of ASD seemed to be assigned much later than parents’ first observation of their child’s developmental deviances. This trend especially increases in cases of mild to moderate ASD or HF-ASD. Since biomarkers are lacking, the encouragement of early detection of mild to moderate ASD symptoms or HF-ASD requires reliable and sensitive instruments.

To help identify ASD, a number of screening instruments have been developed; many are for severely handicapped children with autism [20–21] or for children with ASD at all intelligence levels [22–29]. Moreover, several screening instruments have been developed and validated especially for higher-functioning phenotypes [30–33]. The Autism Spectrum Screening Questionnaire (ASSQ) [30,31] was developed to screen Asperger’s syndrome among school-aged children; however, it was subsequently utilized to screen for other ASDs as well. Although the ASSQ consists of 27 items only, it has shown validity and reliability, with good sensitivity and specificity in clinical settings [31]. In addition, the questionnaire has good internal consistency [34].

As indicated here, the ASSQ has good properties for screening broader phenotypes of autistic traits, but it is standardized for a limited age range (from 7 to 16 years) [30, 31, 35]. A previous study reported parents tended to notice ASD symptoms before their children turned 7, even those with HF-ASD [19]. This suggests ASSQ could detect symptoms of HF-ASD even among children younger than 7. In fact, a previous study used ASSQ with 6-year-old children. [36]. However, examining the scale’s psychometric properties and discriminant validity is important, especially when applying the instrument to an earlier age than determined for its original version.

Thus, the present study examined the ASSQ’s psychometric properties, especially its discriminant validity, when used with 4- to 6-year-old preschool children. The ages of the preschool children vary from country to country. In Japan, children aged 3 to 6 years old are preschool children. More specifically, we used the ASSQ as a part of a medical examination of 5-year-olds in Hirosaki, Japan, to contribute to early detection of ASD, particularly HF-ASD, which is likely to cause maladjustments during children’s school age.

## Methods

### Participants

Two groups of children took part in the present study. The “community” group (*N* = 1390. 814 males, 576 females) was recruited from a Hirosaki Five-year-old Child Developmental Health Check-up Study (2013–2016), which assessed children’s mental health. Hirosaki is a medium-sized city in Aomori Prefecture in northern Japan. The population is approximately 175,000, and the main industry is agriculture. Some questionnaires, mentioned below, were sent to parents of 5-year-old children via the municipal health center after they agreed to participate in the study. They completed the questionnaires and returned them to the municipal health center. The response rate was 74.6%. Basic information on the community group in this study, such as birth weight, gestational age at birth, and families’ household income, was similar to that of Japanese general population [37]. The questionnaire for the 5-year-olds’ medical examination asked if they had previously received an ASD diagnosis and then eliminated those that had been diagnosed earlier.

The “clinical” group (*N* = 154) consisted of children from research centers affiliated with the Graduate School of Medicine, Hirosaki University Medical School, affiliated with the Research Center for Child Mental Development in Hirosaki city. This group consisted of 60 children diagnosed with ASD (ASD group; 48 males, 12 females) and 94 children with non-ASD diagnoses (non-ASD group; 57 males, 27 females). Clinical diagnoses of ASD were confirmed according to the DSM-5 [1] criteria, based on all the available clinical information, by a research team including experienced child psychiatrists and licensed clinical psychologists. To corroborate each diagnosis, we evaluated the severity of autistic symptoms using the Autism Diagnostic Interview Revised (ADI-R) [38, 39] and the Autism Diagnostic Observation Schedule-2 (ADOS-2) [40, 41], administered by research-reliable interviewers. The non-ASD group consisted of 49 children with Attention Deficit Hyperactivity Disorder (ADHD; 52.1%) and 51 children with Developmental Coordination Disorder (DCD; 54.3%). Since their developmental disorders overlapped, the cumulative number of children exceeded 94, which was the total number of non-ASD children.

Table 1 shows the participants’ demographic and clinical characteristics. The proportion of mothers among the informants showed no significant difference. Gender ratio and age did not differ significantly between the community and the clinical groups. Regarding IQ, gestational age at birth, and birthweight, ASD and non-ASD groups showed no significant difference. However, the community group’s gestation at birth was significantly longer than that of the ASD group. Additionally, the community group’s birthweight was significantly heavier than that of both the ASD and non-ASD groups. Regarding families’ household income, ASD and non-ASD groups showed no significant difference, although both of the groups’ household income was significantly lower than that of the community group.

**Table 1:** Characteristics of the study participants.

	Clinical		Community (*n* = 1390)	Analyses (χ^2^ / ANOVA / *t / U*)	Significant pair-wise comparison
	ASD (*n* = 60)	Non-ASD (*n* = 94)			
Male n (%)	42 (70.0%)	57 (60.6%)	814 (58.6%)	χ^2^(2) = 2.7, *p* = 0.201	—
Female n (%)	18 (30.0%)	27 (39.4%)	576 (41.4%)		
informant					
Mother	54 [90%]	91 [96.8%]	1321 [95.0%]	χ^2^(2) = 1.2, *p* = 0.540	—
Father or other care giver	4 [6.7%]	3 [3.2%]	59 [4.2%]		
Missing	2 [3.3%]	0 [0%]	10 [0.7%]		
Average month age (SD)	60.89 (2.16)	60.64 (2.02)	60.53 (1.93)	*F*(2,1541) = 1.12, *p* = 0.328	—
[Range]	[55–72]	[57–71]	[57–68]		
Full-scale IQ	90.08 (13.48)^a^	89.44 (13.77)^b^	—	*t*(138) = 0.263, *p* = 0.793	—
[Range]	[75–123]^a^	[73–122]^b^			
ADI-R total (SD)	34.33 (10.41)^c^	22.90 (8.77)^d^	—	*U* = 173.50, z = 2.90, *p* = 0.003	—
[Range]	[18–51]	[14–44]			
ADOS total (SD)	15.48 (6.12)^c^	6.20 (3.27)^e^	—	*U* = 94.00, z = 2.71, *p* = 0.005	—
[Range]	[4–24]	[2–10]			
ADOS CSS (SD)	7.10 (2.23)	3.40 (2,41)	—	*U* = 93.00, z = 2.71, *p* = 0.006	—
[Range]	[2–10]	[1–6]			
Gestational age at birth					
< 28 week	2 [3.3%]	0 [0%]	7 [0.5%]	χ^2^(2) = 13.2, *p* = 0.001	ASD-NonASD, *p* = 0.103 Community > ASD, *p <* 0.001 NonASD-Community, *p* = 0.831
28 ≤ –37 < week	13 [21.7%]	15 [16.0%]	148 [10.6%]		
37 ≤ –42 < week	43 [71.7%]	72 [76.6%]	1127 [81.1%]		
42 ≤ week	0 [0%]	5 [5.3%]	56 [4.0%]		
Missing	2 [3.3%]	2 [2.1%]	52 [3.7%]		
Birth weight					
< 1000 g	1[1.7%]	0[0%]	2[0.1%]	χ^2^(2) = 15.8, *p* < 0.001	ASD-NonASD, *p* = 1.00 Community > ASD, *p* = 0.010 Community > NonASD, *p* = 0.014
1000–1500 g	2[3.3%]	0[0%]	6[0.4%]		
1500–2500 g	9[15.0%]	16[17.0%]	113[8.1%]		
2500–4000 g	40[66.7%]	71[75.5%]	1191[85.7%]		
> 4000 g	1[1.7%]	1[1.1%]	14[1.0%]		
Missing	7[11.7%]	6[6.4%]	64[4.6%]		
Families’ house hold income					
< JPY 2 milion	7[11.2%]	9[9.6%]	104[7.5%]	χ^2^(2) = 15.9, *p <* 0.001	ASD-NonASD, *p* = 0.687 ASD-Community, *p* = 0.412 Community > NonASD, *p* < 0.001
JPY 2–4 milion	20[33.3%]	44[46.8%]	432[31.1%]		
JPY 4–7 milion	18[30.0%]	31[33.0%]	489[35.2%]		
JPY 7–10 milion	6[10.0%]	3[3.2%]	167[12.0%]		
> JPY 10 milion	2[3.3%]	1[1.1%]	82[5.9%]		
Missing	7[11.7%]	6[6.4%]	116[8.3%]		

ASD, Autism Spectrum Disorder; IQ, Inteligence Quotient; ADI-R, Autism Diagnostic Interview-Revised; ADOS, Autism Diagnostic Observation Schedule; CSS, Calibrated Severity Scores; JPY, Japanese Pay.

^a^ Calculated for 53 participants.

^b^ Calculated for 87 participants.

^c^ Calculated for 21 participants.

^d^ Calculated for 10 participants.

^e^ Calculated for 5 participants.

### Ethics

This study was approved by the Hirosaki University Graduate School of Medicine’s Committee of Medical Ethics in Hirosaki, Japan. Regarding the protection of personal data, this study adhered to both the city and the committee’s information security policies. Written informed consent was obtained from all informants, who were parents or legal guardians of the participating children.

### Questionnaire

#### Autism Spectrum Screening Questionnaire

The ASSQ [30, 31] consists of 27 items rated on a 3-point scale (“0” indicating normal; “1” indicating some abnormality; and “2” indicating definite abnormality). Completing the ASSQ takes approximately 10 minutes, and scores range from 0 to 54. Eleven items cover topics on “social interaction,” while six address “communication problems,” and five refer to “restricted and repetitive behavior.” The remaining five items embrace “motor clumsiness” and other associated symptoms, including “motor and vocal tics.” However, factor analysis of the original Swedish form has not yet been conducted [30, 31]. Subsequent studies have shown the ASSQ has a stable three-factor structure (social difficulties, tics/motor/obsessive-compulsive disorder (OCD), autistic style) [34]. Cut-off scores of 19 or more for the parental-rating version have been recommended for clinical settings in Sweden, to identify ASD among children with normal intelligence or mild mental retardation [30, 31]. Research revealed these scores identified individuals with very high risk of ASD, with specificity of 0.90 and sensitivity of 0.62 for the parental-rating version [31].

In Japan, the ASSQ was translated by the Ministry of Education, Culture, Sports, Science and Technology (MEXT) with the original authors’ permission because, in the early 2000s, easy-to-use screening methods were lacking for high functioning autistic children. For the translation process, first, a researcher specializing in special needs education and a child psychiatrist specializing in developmental disorders each independently conducted a translation. Next, those two translations were reviewed by an expert panel arranged by MEXT to check content validity and finalize the Japanese version of the ASSQ [42], which has been used in clinical settings since 2003, confirming its reliability and validity in Japan [43].

#### Autism Diagnostic Interview-Revised and Autism Diagnostic Observation Schedule

The Japanese versions of the Autism Diagnostic Interview-Revised (ADI-R) [38, 39] and the Autism Diagnostic Observation Schedule (ADOS-2) [40] were used by psychology professionals to examine the ASSQ’s convergent validity. Both instruments collect information from parents and direct observation of children. Items on the ADOS-2 and ADI-R are scored on a 0–3 scale, with higher scores indicating greater impairment. According to ADOS-2 and ADI-R algorithm scoring conventions, all scores of 3 were converted into 2.

The ADI-R yields four domain scores: Qualitative Abnormalities in Reciprocal Social Interaction (Social); Qualitative Abnormalities in Communication (Communication); Restricted, Repetitive, and Stereotyped Patterns of Behavior (RRB-ADI); and Abnormality of Development Evident at or Before 36 Months (Age of Onset), based on past behavior at ages 4 and 5, or ever, and total scores are calculated by summing items within those areas. The total score classifies the participants as autism or non-autism, depending on the algorithm cut-off point.

The ADOS-2 yields two domain scores: Social Affect (SA) and Restricted and Repetitive Behavior (RRB-ADOS) based on direct observation. Those scores are calculated by summing items within those areas. Since the use of Calibrated Severity Scores (CSS) has been shown to be more valid as an indicator of autism severity than the ADOS-2 raw total score [41], CSS scores calculated from raw ADOS-2 scores were used to examine validity in subsequent analyses.

#### Strengths and Difficulties Questionnaire

The Parent-report Strengths and Difficulties Questionnaire (SDQ-P) [44, 45] contains 25 items forming four difficulty subscales: conduct problems; hyperactivity, emotional problems; peer problems; and one favorable subscale, prosocial behavior. To provide concurrent validity of the ASSQ for preschool children, this study examined intercorrelations between the ASSQ and the SDQ-P. Children with high autistic traits are highly likely to have social difficulties, suggesting high correlation between the ASSQ and the SDQ-P [34]. Previous studies that examined the ASSQ’s concurrent validity for school-aged children showed a moderate correlation coefficient of .44 to .66 (*p* < 0.001) [34].

### Analyses

Descriptive statistics were used to explore distribution of preschoolers’ autistic traits that can be measured using ASSQ reported by parents. To examine the discriminant validity of ASSQ items, Kruskal Wallis tests were conducted among the three groups (ASD, non-ASD, and community). Between-group differences were examined by a pair-wise multiple comparison test using rank sums proposed by Dunn [46]. In addition, a polychoric correlation analysis between the rating of ASSQ items (0, 1, 2) and groups (ASD: 1, non-ASD and community group: 0) was performed to examine criterion-related validity of each ASSQ item. Independent samples t-tests were conducted to compare the ASSQ mean scores of school age children with those of preschool children. The results from this study were compared with previous studies’ results for school-aged children [31, 35, 47, 48]. To examine the difference between gender and the ASSQ’s discriminant validity, a two-way Analysis of Variance (Gender [male, female] × Groups [ASD, non-ASD, community]) was conducted on average ASSQ scores. Cronbach’s alpha was calculated to verify the full scale’s internal consistency. Correlation analyses (Pearson’s *r*) were conducted between the ASSQ and the ADI-R, ADOS-2, and SDQ-P to examine convergent and concurrent validity. Receiver Operating Characteristic (ROC) analyses were performed to assess the ASSQ’s discriminant validity. Then, the ROC area under the curve (AUC) was calculated for the ASD group.

PASW Statistics 25 (SPSS) software was used for analyses, excluding subjects with a deficit value for each analysis.

## Results

### Distribution of autistic traits in the population and clinical groups

The ASSQ score distribution for each group is shown in Table 2. In the community group, in which about 20% of children scored 0, and 70% scored 0 to 4, the percentage of children who scored 10 or more was 4.1%, and those who scored 15 or more was 0.8%. The corresponding figures for the ASD group were 0%, 1.7%, 75.0%, and 51.7%, and for the non-ASD group 2.1%, 19.1%, 43.6%, and 20.2%. The highest score was 24 points in the community group. The non-ASD group’s highest score was 26 points, and the ASD group’s was 39 points. The proportion of those who exceeded the existing cutoff point for children of school age [31] was 0.4% in the community group, 10.6% in the non-ASD group, and 38.3% in the ASD group.

**Table 2:** ASSQ percentiles.

ASSQ score	Clinical group				Community group (*N* = 1390)	
	ASD (*N* = 60)		Non-ASD (*N* = 94)			
	Number	Cum percent	Number	Cum percent	Number	Cum percent
0	0	0.0	2	2.1	277	19.9
1	0	0.0	4	6.4	230	36.5
2	0	0.0	4	10.6	200	50.9
3	1	1.7	5	16.0	150	61.7
4	0	1.7	3	19.1	125	70.6
5	2	5.0	5	24.5	83	76.6
6	1	6.7	7	31.9	96	83.5
7	3	11.7	7	39.4	62	88.0
8	2	15.0	5	44.7	51	91.7
9	1	16.7	7	52.1	30	93.8
10	5	25.0	4	56.4	29	95.9
11	1	26.7	4	60.6	18	97.2
12	3	31.7	6	67.0	13	98.1
13	2	35.0	6	73.4	10	98.8
14	5	43.3	3	76.6	4	99.1
15	3	48.3	3	79.8	1	99.2
16	3	53.3	2	81.9	4	99.5
17	3	58.3	3	85.1	1	99.6
18	2	61.7	4	89.4	1	99.6
19	4	68.3	2	91.5	2	99.8
20	4	75.0	4	95.7	1	99.9
21	1	76.7	1	96.8	0	99.9
22	1	78.3	0	96.8	1	99.9
23	0	78.3	1	97.9	0	99.9
24	0	78.3	1	98.9	1	100.0
25	0	78.3	0	98.9	0	100.0
26	1	80.0	1	100.0	0	100.0
27	1	81.7	0	100.0	0	100.0
28	0	81.7	0	100.0	0	100.0
29	2	85.0	0	100.0	0	100.0
30	2	88.3	0	100.0	0	100.0
31	1	90.0	0	100.0	0	100.0
32	0	90.0	0	100.0	0	100.0
33	0	90.0	0	100.0	0	100.0
34	2	93.3	0	100.0	0	100.0
35	0	93.3	0	100.0	0	100.0
36	1	95.0	0	100.0	0	100.0
37	1	96.7	0	100.0	0	100.0
38	1	98.3	0	100.0	0	100.0
39	1	100.0	0	100.0	0	100.0
Total	60	100.0	94	100.0	1390	100.0

ASSQ, Autism Spectrum Screening Questionnaire; ASD, Autism Spectrum Disorder; Cum, Cumlation.

### Means of the ASSQ’s items scores and their differences / polychoric correlation coefficients for ASSQ items

The results of Kruskal-Wallis tests and polychoric correlation coefficients are shown in Table 3. One item only did not discriminate between ASD and community groups. This item was number 1, which says, “Is old-fashioned or precocious.” Polycolic correlation analysis showed weak negative correlation between ASD diagnosis and this item’s score. The result suggested including this item in screening could result in lower discriminant validity of ASD traits for preschool children. The remaining items showed acceptable or good polychoric correlation coefficients, ranging from 0.322 to 0.802. Meanwhile, 19 of 27 items showed differences in scores between the ASD and the non-ASD groups, so that certain discriminant validity was also shown within the clinical group.

**Table 3:** Group differences of ASSQ items and polychoric correlation with ASD diagnosis.

ASSQ items		Clinical group				Community group (*N* = 1390)		Kruscal-Walis		Significant pair-wise comparison	Polychoric correlation^a)^	
		ASD (*N* = 60)		Non-ASD (*N* = 94)								
		Mean	*SD*	Mean	*SD*	Mean	*SD*	*χ*^*2*^	*p-value*		Coefficients	*p-value*
1	Old-fashioned or precocious	0.283	0.555	0.426	0.558	0.477	0.641	6.19	0.045	Community > ASD	-0.186	0.008
2	Eccentric professor	0.250	0.541	0.053	0.226	0.072	0.307	19.67	< 0.001	ASD > Non-ASD, Community	0.322	< 0.001
3	Lives in own world	0.633	0.823	0.426	0.680	0.183	0.454	47.15	< 0.001	ASD, Non-ASD > Community	0.401	< 0.001
4	Accumulate facts	0.683	0.725	0.500	0.668	0.264	0.495	42.13	< 0.001	ASD, Non-ASD > Community	0.372	< 0.001
5	Literal understanding	0.983	0.748	0.638	0.686	0.398	0.580	53.83	< 0.001	ASD > Non-ASD > Community	0.428	< 0.001
6	Robot-like language	0.600	0.741	0.309	0.529	0.056	0.248	177.90	< 0.001	ASD > Non-ASD > Community	0.666	< 0.001
7	Idiosyncratic words	0.600	0.807	0.447	0.666	0.150	0.412	67.76	< 0.001	ASD, Non-ASD > Community	0.434	< 0.001
8	Different voice/speech	0.450	0.723	0.309	0.605	0.097	0.339	56.65	< 0.001	ASD, Non-ASD > Community	0.444	< 0.001
9	Involuntary sounds	0.533	0.747	0.223	0.490	0.058	0.258	115.67	< 0.001	ASD > Non-ASD > Community	0.616	< 0.001
10	Uneven abilities	1.033	0.843	0.479	0.600	0.196	0.454	131.76	< 0.001	ASD > Non-ASD > Community	0.618	< 0.001
11	No social fit in language	1.017	0.725	0.642	0.736	0.169	0.390	220.53	< 0.001	ASD > Non-ASD > Community	0.695	< 0.001
12	Lacks empathy	0.617	0.666	0.283	0.551	0.035	0.196	323.66	< 0.001	ASD > Non-ASD > Community	0.756	< 0.001
13	Naïve remarks	0.917	0.743	0.691	0.656	0.239	0.454	129.80	< 0.001	ASD, Non-ASD > Community	0.556	< 0.001
14	Deviant style of gaze	0.317	0.624	0.258	0.550	0.059	0.262	59.25	< 0.001	ASD, Non-ASD > Community	0.448	< 0.001
15	Fails to make friend	0.750	0.704	0.436	0.597	0.111	0.323	167.69	< 0.001	ASD > Non-ASD > Community	0.659	< 0.001
16	Sociable on own terms only	0.817	0.770	0.447	0.650	0.117	0.335	157.60	< 0.001	ASD > Non-ASD > Community	0.659	< 0.001
17	Lacks best friend	0.483	0.701	0.149	0.387	0.019	0.151	224.44	< 0.001	ASD > Non-ASD > Community	0.764	< 0.001
18	Lacks common sense	0.800	0.708	0.489	0.600	0.079	0.277	271.88	< 0.001	ASD > Non-ASD > Community	0.725	< 0.001
19	Poor at games, own goals	0.900	0.775	0.596	0.628	0.096	0.309	284.26	< 0.001	ASD > Non-ASD > Community	0.724	< 0.001
20	Clumsy	0.817	0.792	0.550	0.516	0.044	0.208	287.51	< 0.001	ASD > Non-ASD > Community	0.712	< 0.001
21	Involuntary movements	0.717	0.825	0.202	0.454	0.038	0.220	242.73	< 0.001	ASD > Non-ASD > Community	0.760	< 0.001
22	Compulsory repetitive	0.750	0.816	0.372	0.656	0.055	0.243	222.01	< 0.001	ASD > Non-ASD > Community	0.736	< 0.001
23	Insists on no change	0.817	0.813	0.319	0.553	0.112	0.346	138.49	< 0.001	ASD > Non-ASD > Community	0.641	< 0.001
24	Idiosyncratic attachment	0.864	0.819	0.447	0.666	0.202	0.466	86.04	< 0.001	ASD > Non-ASD > Community	0.532	< 0.001
25	Bullied by other children	0.283	0.490	0.213	0.484	0.047	0.211	70.69	< 0.001	ASD, Non-ASD > Community	0.509	< 0.001
26	Unusual facial expression	0.283	0.640	0.106	0.343	0.019	0.153	74.62	< 0.001	ASD > Non-ASD > Community	0.592	< 0.001
27	Unusual posture	0.350	0.685	0.138	0.454	0.015	0.133	123.96	< 0.001	ASD > Non-ASD > Community	0.699	< 0.001

ASSQ, Autism Spectrum Screening Questionnaire; ASD, Autism Spectrum Disorder.

^a)^ Rating of each item(0,1,2) × Group (ASD group = 1, non-ASD and community groups = 0).

### Means of the ASSQ scores and their differences

Independent samples *t*-tests were conducted to compare the mean ASSQ scores of school age children with those of preschool children. This study’s results were compared with those of previous studies [31, 35, 47, 48]. The means of ASSQ scores in the ASD, non-ASD, and community groups were 17.62 (*SD* = 9.13), 10.06 (*SD* = 6.09), and 3.40 (*SD* = 3.34). In the ASD group, the result of comparing the mean score obtained in this study with scores from school age studies (Ehlers et al. [31]: *M* = 25.1, *SD* = 7.3, *N* = 34; Mattila et al. [47]: *M* = 24.30, *SD* = 8.50, *N* = 47; Guo et al. [48]: *M* = 25.3, *SD* = 9.2, *N* = 94), showed significant differences (Ehlers et al. [31]: *t* (92) = 4.09, *p* < 0.001, *d* = 0.88; Mattila et al. [47]: *t* (105) = 3.87, *p* < 0.001, *d* = 0.75; Guo et al. [48]: *t* (92) = 4.09, *p* < 0.001, *d* = 0.84).

Meanwhile, in the community group, the result of comparison with previous studies at school age (Posserud et al. [35]: *M* = 3.29, *SD* = 4.49, *N* = 6229; Mattila et al. [47]: *M* = 2.00, *SD* = 4.49, *N* = 3565; Guo et al. [48]: *M* = 5.2, *SD* = 6.6, *N* = 120), showed no significant difference between this study and the study by Posserud et al. [35] (*t* (7617) = 0.860, *p* = 0.390, *d* = 0.03). However, there were significant differences between this study and the study by Mattila et al. [47] (*t* (4953) = 12.81, *p* < 0.001, *d* = 0.38) and the study by Guo et al. [48] (*t* (1508) = 5.11, *p* < 0.001, *d* = 0.49).

Table 4 presents the mean scores described separately by gender, their standard deviations, and ranges of ASSQ scores for each group, which shows, as a result of two-way ANOVA (Gender [male: female] × Groups [ASD: non-ASD: community]), the main effects of group and gender were significant, while interaction was not.

**Table 4:** Two-way analysis of variance for ASSQ total scores.

gender	Clinical		Community (*N* = 1390)	Two-way ANOVA gender (male: female)×group (ASD: NonASD:Community)			
	ASD (*N* = 60) Mean (SD) [Range]	Non-ASD (*N* = 94) Mean (SD) [Range]	Mean (SD) [Range]	interaction	gender	group	
							Significant pair-wise comparison
Male	18.12 (9.17) [5–39]	10.33 (6.49) [0–26]	3.66 (3.63) [0–24]	*F*(2, 1538) = 0.43 *p* = 0.650 η_p_^2^ = 0.001	*F*(1, 1538) = 4.44 *p* = 0.035 η_p_^2^ = 0.003	*F*(2, 1538) = 400.69 *p* < 0.001 η_p_^2^ = 0.343	ASD > NonASD > Community *p* < 0.001
Female	16.44(9.18) [3–37]	9.65(5.47) [1–20]	3.04(3.40) [0–19]				ASD > NonASD > Community *p* < 0.001

ASSQ, Autism Spectrum Screening Questionnaire; ANOVA, Analysis of variance; ASD, Autism Spectrum Disorder.

The main effect of group and gender was significant; therefore, we performed a simple main effect test. Concerning gender factors in the ASD group, there was no significant difference in scores between males and females (*F*(1,1538) = 2.246, *p* = 0.134, η_p_^2^ = 0.001). In addition, in the non-ASD group, there was no significant difference in scores between males and females (*F*(1,1538) = 0.669, *p* = 0.414, η_p_^2^ < 0.001). However, in the community group there was significant difference in scores between males and females (*F*(1,1538) = 8.19, *p* = 0.004, η_p_^2^ = 0.005).

The simple main effect of group was significant in males (*F*(2,1538) = 325.80, *p* < 0.001, η_p_^2^ = 0.298) = 350.89, *p* < 0.001, η_p_^2^ = 0.313) and females (*F*(2,1538) = 142.217, *p* < 0.001, η_p_^2^ = 0.156). As a result of multiple comparisons by the Bonferroni method, ASSQ scores were significantly higher in the ASD group than in the non-ASD and community groups in both males and females. In the non-ASD group, ASSQ scores were significantly higher than the community group’s scores in both males and females.

### Internal consistency

Both clinical and community groups showed good internal consistency for the ASSQ, with Cronbach’s alpha of 0.844–0.881 [95% CI:0.808–0.930]. The details of the results are presented in Table 5.

**Table 5:** Internal consistency of the ASSQ.

		Clinical group			Community group (*N* = 1390)
		All	ASD (*N* = 60)	Non-ASD (*N* = 94)	
ASSQ	*α* [95% CI]	0.876 [0.812–0.928]	0.881 [0.816–0.924]	0.874 [0.808–0.930]	0.844 [0.819–0.868]

ASSQ, Autism Spectrum Screening Questionnaire; ASD, Autism Spectrum Disorder; CI, Confidence Interval.

### Convergent and concurrent validity

To examine the ASSQ’s convergent validity, correlation analysis was conducted with ADI-R [38, 39] and ADOS-2 [40], which are regarded as gold standards of ASD measurement. The ASSQ scores were correlated with ADI-R total scores to a strong degree. Among ADI-R subscales, social domain showed the highest correlation with the ASSQ. ADOS-2 CSS scores [41] were also correlated with the ASSQ scores to a moderate degree. Both of the ADOS-2 subscales showed moderate correlation with ASSQ.

The ASSQ scores yielded high correlation with SDQ-P total difficulties scores. The correlations with the SDQ-P prosocial score were negative because it is a strengths scale, whereas the ASSQ items are scored as difficulties. The ASSQ score correlated the highest with the SDQ-P peer problems subscale. The details of the results are presented in Table 6.

**Table 6:** Correlation with ADI-R, ADOS, SDQ.

Pearson correlation	*r*	*p*
ADI-R		
Total	0.631^a^	< 0.001
Social	0.607^a^	< 0.001
Communication	0.413^a^	0.017
RRB-ADI	0.338^a^	0.054
Age of onset	0.367^a^	0.036
ADOS		
CSS	0.478^b^	0.028
Social affect	0.489^b^	0.008
RRB-ADOS	0.424^b^	0.025
SDQ total		
Total difficulties	0.570^c^	< 0.001
Peer	0.467^c^	<0.001
Emotinal	0.346^c^	< 0.001
Hyperactivity	0.439^c^	< 0.001
Conduct	0.357^c^	< 0.001
Prosocial	‐0.263^c^	< 0.001

ADI-R, Autism Diagnostic Interview-Revised; ADOS, Autism Diagnostic Observation Schedule; SDQ, Strengths and Difficulties Questionnaire; RRB, Restricted, Repetitive and Stereotyped Patterns of Behavior; CSS, Calibrated Severity Scores.

^a^Calculated for 31 participants,

^b^Calculated for 31 participants,

^c^Calculated for 1544 participants.

### Discriminant validity

**Fig 1:**
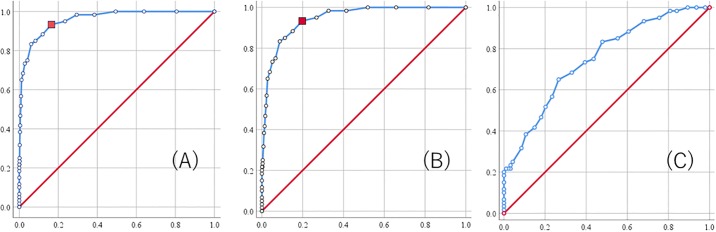
Receiver Operating Characteristic curve for the ASSQ scores. (A) ROC curve for ASD and community children. (B) ROC curve for ASD and community + non-ASD children. (C) ROC curve for ASD and non-ASD children.

ROC analyses were performed to assess the ASSQ’s discriminant validity in distinguishing the ASD from the non-ASD and community groups. In addition, ROC analyses were conducted on combined non-ASD and community group data (community + non-ASD) to examine the ASSQ’s identification accuracy for the combined group. For the AUC obtained from ROC results, 0.60–0.75 is said to indicate a moderate-level discriminant accuracy, 0.75–0.90 is a good level, 0.90–0.97 is a very good level, and 0.97–1.00 is an optimum level [49]. ROC analyses revealed the scale’s ability to distinguish children with ASD against community children with an area of 0.960 under the curve (95%CI: 0.939–0.981) (Fig 1A). The ASSQ’s identification accuracy for the non-ASD + community group did not decrease markedly compared to the community group alone, and accuracy was actually shown to have maintained a certain level (AUC = 0.946, 95% CI = 0.923–0.970) (Fig 1B). On the other hand, discriminatory power within clinical groups (AUC = 0.749, 95% CI = 0.671–0.826) (Fig 1C) was lower than the ASSQ’s discriminant power in distinguishing ASD from community children.

In the community group and community + non-ASD sample, a cut-off score of ≥ 7 indicated the best balance between sensitivity (93%) and specificity (community = 84%, community + non-ASD = 80%). The corresponding score of the non-ASD sample was 14; however, adequate results were not obtained for sensitivity (65%) and specificity (73%). Table 7 shows more detailed statistics of ASSQ scores.

**Table 7:** Inpact of ASSQ cutoff score on sensitivity and specificity.

Cutoff Score	SE	vs Community		vs Non-ASD		vs Community + NonASD	
		SP	SE+SP	SP	SE+SP	SP	SE+SP
1	1.00	0.19	1.19	0.02	1.02	0.18	1.18
2	1.00	0.36	1.36	0.06	1.06	0.34	1.34
3	1.00	0.51	1.51	0.11	1.11	0.48	1.48
4	0.98	0.62	1.60	0.16	1.14	0.59	1.57
5	0.98	0.71	1.69	0.19	1.17	0.67	1.66
6	0.95	0.77	1.72	0.24	1.19	0.73	1.68
**7**	0.93	0.84	1.77	0.32	1.25	0.80	1.75
8	0.88	0.88	1.76	0.39	1.28	0.85	1.73
9	0.85	0.92	1.77	0.45	1.30	0.89	1.74
10	0.83	0.94	1.77	0.52	1.35	0.91	1.75
11	0.75	0.96	1.71	0.56	1.31	0.93	1.68
12	0.73	0.97	1.71	0.61	1.34	0.95	1.68
13	0.68	0.98	1.66	0.67	1.35	0.96	1.64
14	0.65	0.99	1.64	0.73	1.38	0.97	1.62
15	0.57	0.99	1.56	0.77	1.33	0.98	1.54
16	0.52	0.99	1.51	0.80	1.31	0.98	1.50
AUC		0.960		0.749		0.946	
95%CI		0.939–0.981		0.671–0.826		0.923–0.970	

ASSQ, Autism Spectrum Screening Questionnaire; SE, sensitivity; SP, specificity; AUC, area under the curve; CI, confidence interval.

## Discussion

### Summary of findings

This study examined the ASSQ’s psychometric properties among preschool children recruited from the community and clinical settings. The results showed, as in the case of school-aged children, the ASSQ had reliability and validity as a screening instrument in community screening for preschool children. On the contrary, a significant difference between the clinical samples was indicated in the ASSQ’s mean score. Although identification validity was partially demonstrated, ROC analyses results did not show adequate identification validity for use as a screening tool in clinical settings.

### Reliability

The Cronbach’s alpha coefficient indicated sufficient internal consistency among all the groups. In a study with a community sample of school-aged children (ages 7 to 9) [34], the Cronbach’s alpha coefficient was 0.86: A similar level of internal consistency for the ASSQ for preschool children was also found. The internal consistency of the ASD screening instrument, which is similar to the ASSQ applied to preschool children, was 0.99 for the Autism Spectrum Quotient: Children’s Version (AQ-Child) [23]; 0.891–0.942 for the preschool version of the Social Responsiveness Scale (SRS-P) [50]; and 0.839 for the Childhood Autism Spectrum Test (CAST) [51]. As a coefficient, internal consistency tends to increase as the number of items on a scale increases. As such, a scale needs to be compared with another scale with a similar number of items. Of the three scales mentioned above, the AQ-Child had 50 items [23], the SRS-P had 65, [50] and the CAST had 37 [51]. Even with regard to the number of items, the ASSQ’s internal consistency is believed to be satisfactory to a certain level as a screening instrument for the same age group.

#### Convergent and concurrent validity

The ASSQ score indicates a moderate-to-large level correlation with the ADI-R and the ADOS-2, which are said to be gold standards for ascertaining ASD characteristics, and it has convergent validity when used for preschool children. Since validation of the correlation coefficient between the ASSQ and ADI-R and between the ASSQ and ADOS-2 had not been ascertained with school-aged children, this study cannot easily be compared with others. However, a study that conducted SRS with preschool children [52] found a correlational coefficient with a level similar to this study’s (ADI-R: *r* = 0.63; ADOS-2: *r* = 0.48). Therefore the ASSQ had convergent validity to the same degree as other scales that target preschool children.

In addition, not only was a strong positive correlation found between the ASSQ score and the SDQ-P total difficulties score, but content validity was also confirmed. Between the subscales of the ASSQ and SDQ-P, an overall moderate-level correlation was found. The item that showed the highest correlation within subscales was Peer Problems. This shows the scale’s content validity is satisfactory since ASD’s core disorder is social difficulty.

### Difference between preschool and school-aged children’s ASSQ score

This study’s results indicate possibly preschool ASD children’s ASSQ score is lower than their average scores in previous studies targeting school-aged children. Items with lower scores compared with other items are item number 1 “Old-fashioned or precocious” (Mean = 0.283) which did not demonstrate identification validity, and item number 2 “Eccentric professor” (Mean = 0.250). Originally, ASSQ is a screening tool especially targeted to Asperger’s syndrome that is not noticeable delay in language acquisition, and item number 1 is considered to capture the precocious of knowledge and speech of Asperger’s syndrome. However, a wide range exists in language acquisition for HF-ASD, from precocious speech to severe speech onset delay at early age [53]. In this study, the ASD group consisted of preschool children with HF-ASD (IQ> 70), but the level of language development was not taken into consideration in constructing this group. For these reasons, it was possibly difficult to categorically describe ASD group children as precocious. Additionally, although item number 2 describes a trait of HF-ASD, which is significant knowledge of limited areas such as calendars and timetables, this trait becomes gradually noticeable after starting school and through school age. The score of this item of the ASD group was significantly higher than that of the community group, so it is assumed that this trait was seen somewhat in the ASD group, but it is considered that it was not as noticeable as school-aged children. In this way, possibly, this study’s items representing traits that become gradually noticeable through school age mainly obtained low scores, leading to the significant difference (d = 0.75–0.88) in the overall mean score compared to school-aged children’s mean score. However, the polychoric correlation of presence or absence of an ASD diagnosis and the ASSQ scores show that item has significant discriminatory ability with the exception of item number 1; therefore, the tendency of lower scores for ASD children does not necessarily limit the ASSQ’s applicability for preschool children.

On the contrary, comparison of mean scores between the community group of school-aged children and this study did not show a consistent result. However, from the viewpoint of effect size, which measures difference between mean scores, the effect size obtained between the community group and previous studies was generally small (d = 0.03–0.49), suggesting the ASSQ performed at the same level as school-age studies.

### Gender differences for preschool children

The community group showed a gender difference in ASSQ scores, the clinical group did not. Similar results—gender difference in the community group [35] and no gender difference in ASD children [36, 47]—were shown in previous studies targeting school-aged children. As noted by Mattila et al. [47], however, there were a similarly limited number of female samples (n = 18) in this study, and score variations in ASD female samples were large. For these reasons, non-existence of gender difference in ASD children should be concluded with care. Conversely, the tendency to see even a slight difference as significant due to the large number of samples should be considered with regard to community samples.

### Discriminant validity and examination of optimum cut-off score

With reference to score distribution in the community group, 19.9% of the participants had a score of 0 points, 50.9% of the participants obtained 3 points or less, and 91.7% of the participants obtained 8 points or less. This result is similar to those of the school-aged community sample [35]. In the community sample (N = 6,229), 25.8% of the children scored 0 points [35]. When item number 1, which had a negative relationship with ASD diagnosis, was eliminated, the percentage of children scoring 0 points increased from 19.9% to 27.7%. The latter result is closer to previous studies’ score distributions [35]. This result shows at the possibility of item number 1 falsely and highly assessing the ASD trait; therefore, it should be handled carefully. In the clinical group, 52.1% of the non-ASD group received 9 points, and 53.3% of the ASD group received 16 points: This indicates clearly the community group’s scores were distributed more in the lower point range. The ANOVA results showed the difference in scores within the group was significant for both males and females: the effect size showed such differences were large [54]. Furthermore, a multiple comparison shows scores were the highest in the order of ASD > non-ASD > community groups for both males and females, indicating a certain level of discriminant validity of ASSQ for preschool children.

In this study, the AUC for the community sample was 0.960 (95% CI: 0.939–0.981). Additionally, ROC analyses for community + non-ASD data showed the AUC did not decrease compared with the community alone (AUC = 0.946, 95% CI = 0.923–0.970), confirming that the ASSQ has a very good to optimum level of discriminant validity [49] as a screening tool for preschool children on a community setting. Furthermore, the AUC of the ASSQ that targeted school children from 7 to 9 years old was 0.90–0.97 [55, 56], indicating it had similar discriminant validity among preschool children. The AUC of the scale targeting preschool children, similar to the ASSQ, was 0.99 (95% CI: 0.98–0.99) for AQ-Child [23]; 0.874 (95% CI: 0.810–0.939) for SRS-P [50]; and 0.931 for CAST [51]. The ASSQ’s discriminant ability was inferred as satisfying a certain level when compared with the population-based screening instrument for the same age group.

Previous studies presented a cut-off value of 7–12 points (sensitivity = 0.91–0.96, specificity = 0.77–0.93) when using the ASSQ in a community setting that emphasizes sensitivity [48, 55, 56]. This study showed the two cut-offs of 7 and 10 are scores with the highest total scores of sensitivity and specificity, and for which the scale can be used most effectively, for both community and community + non-ASD. In the community sample, the cut-off value of 7 identified 93% of participants diagnosed with ASD according to the DSM-5 with 84% specificity, while the cut-off score of 10 identified 83% of participants diagnosed as having ASD, but with higher specificity (94%). In conclusion, we suggest the use of a cut-off score of 7 in the ASSQ for preschool children in community settings, because, in a primary screening tool, prioritizing sensitivity is necessary to minimize overlooking children possibly having ASD. Thus, based on this study, it may be necessary to set the cut-off value slightly lower when ASSQ is applied to preschool children. However, as shown in the analysis of the community + non-ASD sample, specificity drops in samples including relatively many neurodevelopmental disorders such as ADHD and DCD. This indicates the necessity of fully examining more false positives through clinical examinations. Thus, cut-off scores depending on the scale’s intended use should be established.

Based on the current study using the clinical setting, an optimal cut-off score with high sensitivity and high specificity could not be established. When sensitivity was high, specificity was low, and vice versa. Additionally, a low AUC (0.749, 95% CI: 0.671–0.826) demonstrated the ASSQ for preschool children does not work well in a clinical setting. As presented in Table 3, ASSQ items showed significant discriminant validity for 26 of 27 items in the community group, but in the non-ASD group, 19 items had discriminant validity. Presumably, the AUC significantly decreased because of these items’ impact. As mentioned previously, the non-ASD group in this study comprised children with ADHD and DCD. Some children with ADHD, but not with severe ASD have scores that surpass the diagnostic cut-off value even if an ASD trait ascertaining method (e.g., ADOS-2) much stricter than a questionnaire survey is used [57]. A similar limitation was found in this study. DCD is a neurodevelopmental disorder in which the core is difficulties involving acquisition and execution of coordinated movements [1]. The study that explored the ASSQ’s factor structure found factors that include difficulties in movement [34]. With reaction of such items, certain items’ (e.g., numbers 19 and 20) discriminant ability was believed to have been lower than in the community sample. Thus, while simultaneously maintaining sensitivity and specificity in a clinical setting was difficult, previous study on school-aged children also reported difficulty establishing a parental evaluation cut-off score with high sensitivity and specificity in a clinical setting [56]. The clinicians should be reminded that the ASSQ is a screening instrument, not a diagnosing instrument. For limitations to parent evaluations in a clinical setting, Mattila et al. [56] recommended using the total score of the parent and the teacher evaluation. In that study, by using the total score of the teacher rating and the parent rating, reported improvement the accuracy of identification for high to medium risk samples [56]. Furthermore, identification accuracy considerably improved when using the total parent and teacher scores, rather than one score only, in community screening [55, 56], in addition to the clinical setting, suggesting the importance of multifaceted evaluation.

### Limitations and perspectives

This study has several limitations. First, an important limitation is the non-ascertainment of community high-scorers. In the community group, the questionnaire for the 5-year-old medical examination asked if they had received an ASD diagnosis before, and eliminated those previously diagnosed with ASD; however, there is still a possibility that a certain number of undiagnosed ASD were included. Especially for the children in the high-score zones in the community group, there is a relatively high chance that they would be diagnosed with ASD by a clinical examination, possibly falsely lowering the specificity in this study. Second, there were differences in several points between this study and the previous study targeting school-aged children. Part of this is considered to be the result reflecting a difference in the age factor between preschool and school-aged children. However, a confounding cultural factor is also a possibility. We were not able to discuss this point because mean scores and cut-off scores in domestic studies for school-aged children were not shown [43]. Third, this study was administered only in a medium-sized city in Japan, thereby limiting its generalizability to other regions. Fourth, we did not consider comorbidities such as ADHD, which could be diagnosed with ASD in the DSM-5. Fifth, the Japanese version of ASSQ has been translated by experts, and reliability and validity have been confirmed in Japanese school-aged children, but strict back-translation [58] has not been done. Finally, previous studies have shown that using total points from both parent-rated and teacher-rated ASSQ indicates the most favorable identification accuracy. However, since no teacher-rated ASSQ was used for this study, this point needs to be examined. Additionally, examining to what degree the teacher-rated ASSQ affects identification accuracy when applying it to preschool children is important.
