# Correction: Adaptation of the Autism Spectrum Screening Questionnaire (ASSQ) to preschool children

**DOI:** 10.1371/journal.pone.0203254

**Published:** 2018-08-27

**Authors:** Masaki Adachi, Michio Takahashi, Nobuya Takayanagi, Satomi Yoshida, Sayura Yasuda, Masanori Tanaka, Ayako Osato-Kaneda, Manabu Saito, Michito Kuribayashi, Sumi Kato, Kazuhiko Nakamura

The second affiliation for the eleventh author is incorrect. Kazuhiko Nakamura is not affiliated with #4 but with #3 Department of Neuropsychiatry, Graduate School of Medicine, Hirosaki University, Hirosaki, Aomori, Japan.
